# Newborn care practices at home and in health facilities in 4 regions of Ethiopia

**DOI:** 10.1186/1471-2431-13-198

**Published:** 2013-12-01

**Authors:** Jennifer A Callaghan-Koru, Abiy Seifu, Maya Tholandi, Joseph de Graft-Johnson, Ephrem Daniel, Barbara Rawlins, Bogale Worku, Abdullah H Baqui

**Affiliations:** 1 International Center for Maternal and Newborn Health, Department of International Health, Johns Hopkins Bloomberg School of Public Health, Baltimore, MD, USA; 2 Maternal and Child Health Integrated Program, Addis Ababa, Ethiopia; 3 Jhpiego, Baltimore, MD, USA; 4 Maternal and Child Health Integrated Program, Washington, DC, USA; 5 School of Medicine, Department of Pediatrics and Child Health, Addis Ababa University, Addis Ababa, Ethiopia

## Abstract

**Background:**

Ethiopia is one of the ten countries with the highest number of neonatal deaths globally, and only 1 in 10 women deliver with a skilled attendant. Promotion of essential newborn care practices is one strategy for improving newborn health outcomes that can be delivered in communities as well as facilities. This article describes newborn care practices reported by recently-delivered women (RDWs) in four regions of Ethiopia.

**Methods:**

We conducted a household survey with two-stage cluster sampling to assess newborn care practices among women who delivered a live baby in the period 1 to 7 months prior to data collection.

**Results:**

The majority of women made one antenatal care (ANC) visit to a health facility, although less than half made four or more visits and women were most likely to deliver their babies at home. About one-fifth of RDWs in this survey had contact with Health Extension Workers (HEWS) during ANC, but nurse/midwives were the most common providers, and few women had postnatal contact with any health provider. Common beneficial newborn care practices included exclusive breastfeeding (87.6%), wrapping the baby before delivery of the placenta (82.3%), and dry cord care (65.2%). Practices contrary to WHO recommendations that were reported in this population of recent mothers include bathing during the first 24 hours of life (74.7%), application of butter and other substances to the cord (19.9%), and discarding of colostrum milk (44.5%). The results suggest that there are not large differences for most essential newborn care indicators between facility and home deliveries, with the exception of delayed bathing and skin-to-skin care.

**Conclusions:**

Improving newborn care and newborn health outcomes in Ethiopia will likely require a multifaceted approach. Given low facility delivery rates, community-based promotion of preventive newborn care practices, which has been effective in other settings, is an important strategy. For this strategy to be successful, the coverage of counseling delivered by HEWs and other community volunteers should be increased.

## Background

A systematic analysis of progress toward Millennium Development Goal 4 indicates that mortality among children under five years old has dropped worldwide from 11.9 million deaths per year in 1990 to 7.7 million deaths in 2010 [1]. Most of the decline has been in older infants and children ages 1 to 4, and consequently neonatal deaths now account for a greater proportion of under-five deaths [1]. An estimated 3.1 million neonates die each year globally, and 99% of these deaths occur in low-income countries [2]. Neonatal deaths represented an estimated 40% of under-five deaths in 2010 [3]. Although neonatal mortality rates are also decreasing globally, Africa is experiencing much slower declines than other regions [2]. As a result of insufficient progress, there have been increasing calls for action to address newborn survival [4-6].

Promotion of essential newborn care practices is one strategy for improving newborn health outcomes. The World Health Organization has defined essential newborn care to include clean delivery and clean cord care, thermal protection, early and exclusive breastfeeding, initiation of breathing and resuscitation, eye care, immunization, care for the low birth weight newborn, and management of newborn illness [7], and has developed a training course for health workers. In settings where a majority of births take place at home without a skilled attendant and care seeking rates are low, preventive interventions included in essential newborn care may also be promoted at the community level [8-12]. For example, promotion of preventive behaviors through home visits by community health workers has been shown to improve key newborn care practices such as early initiation of breastfeeding, skin-to-skin contact and delayed bathing to prevent hypothermia, and clean care of the umbilical cord [10]. However, recommended newborn care practices may conflict with local beliefs and practices that are risk-enhancing [13-15]. It is therefore critical to understand the existing newborn care practices in order to adapt behavior change interventions to be successful [16].

Ethiopia is one of the ten countries with the highest number of neonatal deaths globally, with an estimated 122,000 newborn deaths per year [9]. Close to 90% of deliveries in Ethiopia take place at home, and attendance at antenatal care and postnatal care are also inadequate [17]. As a result of low facility delivery rates, the Federal Ministry of Health (FMOH) in Ethiopia established a policy for the delivery of maternal and neonatal health interventions through prenatal and postnatal home visits made by health extension workers (HEWs). There is very limited information about newborn care practices in Ethiopia because many key indicators are not currently measured by routine surveys like the Demographic and Health Survey. Here we report results of a baseline survey conducted as part of an evaluation of the promotion of newborn care practices and kangaroo mother care by Health Extension workers. This study aims to describe newborn care practices reported by recently-delivered women (RDWs) across four regions of Ethiopia, and is to our knowledge the first study in Ethiopia to compare newborn care practices between home births and facility deliveries [18].

## Methods

### Study setting

The Federal Democratic Republic of Ethiopia is the second most populous country in Africa with a population of 85 million. The population is growing at a rate of 2.6% per year [19], and the total fertility rate is estimated at 4.8 children per woman [17]. According to the 2007 census, 84% of the population lives in rural areas where the primary occupation is farming, making Ethiopia one of the least urbanized countries in the world [20]. Ethiopia also has the tenth largest land area in Africa, with diverse geography and peoples and over 80 spoken languages.

This study includes the regions of Oromia, Tigray, Amhara, and Southern Nations, Nationalities, and People (SNPP), which are supported by the United States Agency for International Development's Maternal and Child Health Integrated Program in a pilot implementation of community-based newborn and kangaroo mother care promoted by Health Extension Workers. These four regions were chosen for the pilot program because they account for more than 85 percent of the country’s total population [19] and represent the diverse cultural and linguistic differences of the many ethnic groups in the country. Table 1 presents demographic and health indicators for these four regions.

**Table 1:** Characteristics of study regions

**Indicators**	**National**	**Amharra**	**Oromia**	**SNNP**	**Tigray**
***Demographic indicators***					
Population*	73,918,505	17,214,056	27,158,471	15,042,531	4,314,456
Proportion urban population*	16%	13%	12%	10%	20%
Proporiton of men who are engaged in agriculture	73%	80%	77%	77%	71%
Total fertility rate (per woman)	4.8	4.2	5.6	4.9	4.6
Proportion of women who are literate	38%	36%	38%	31%	45%
***Mortality rates***					
Under-five mortality (per 1,000 live births)	88	108	12	116	85
Infant mortality (per 1,000 live births)	59	76	73	78	64
Neonatal mortality (per 1,000 live births)	37	54	40	38	44
***Maternal and child health services indicators***					
Proportion of pregnant women receiving antental care from a skilled provider	57%	59%	61%	59%	35%
Proportion of deliveries at a skilled facility	10%	10%	8%	6%	12%
Proportion of women with a postnatal check up in first 2 days after birth	7%	5%	5%	6%	13%
Proportion of children (age 12–23 months) who received all basic vaccinations by twelve months	24%	26%	16%	24%	59%

Sources: 2011 DHS Survey; *2007 National Census.

The Ethiopian Federal Ministry of Health provides primary health services free of charge through primary hospitals (1 per 60,000-100,000 population), health centers (1 per 15,000- 25,000) and health posts (1 per 5,000 people). In 2003, in order to extend primary care access to rural areas, the government established a new cadre of workers, known as Health Extension Workers (HEWs), to provide basic health care from rural health posts [21]. The package of services provided by health extension workers includes environmental health promotion, family planning, immunization, and maternal and child health services [21]. HEWs are typically young women with at least a grade 10 education and receive one-year of training before deployment to a Health Post in their community [22]. More than 34,000 HEWs currently provide basic health services from 15,666 health posts across the country. Supporting the HEWs is the volunteer Health Development Army, composed of approximately 1 household with a model woman networked with 5 other households, who mobilize the community and provide health education.

Despite Ethiopia’s achievements to improve access to maternal, newborn, and child health services, accelerated progress is needed for the country to achieve Millennium Development Goal 4 [23], particularly in the area of newborn health. Currently 1 in every 27 Ethiopian children dies within his or her first month of life [7]. Nationally, neonatal deaths account for 42% of under-five deaths [17] and the primary causes for newborn death include birth asphyxia (30%), sepsis (24%), prematurity (23%), and pneumonia (8%) [3]. The neonatal mortality rates in the four regions included in this study range between 38 per 1,000 in SNNP to 54 per 1,000 live births in Amharra (Table 1).

Routine health services for mothers and newborns are severely underutilized across Ethiopia. According to the DHS 2010, only 34% of women receive any antenatal care from a skilled provider, 10% of births take place at a health facility, and 7% of women receive a postnatal check up within the first two days of birth [17]. Reasons reported for low utilization of maternal health services in Ethiopia include lack of perceived need, distance to services, costs of services, negative experiences with or perceptions of quality of care at facilities, and preference for traditional birthing practices [24,25].

### Survey design and sampling

This article provides the results from a cross-sectional household survey of newborn care practices conducted to establish a baseline for a study to assess the feasibility of recently delivered women (RDWs) adopting kangaroo mother care (KMC) when promoted by HEWs and other health service providers. The study site included the catchment areas of 10 health centers in four regions—Tigray, Oromiya, Amhara, and SNNPR—that are participating in the pilot. Facility-based KMC was established at these ten health centers and facility staff received essential newborn care training prior to the baseline survey. However, the survey took place before the training of Health Extension Workers on community-level newborn care and kangaroo mother care promotion.

We sampled 30 census enumeration areas (EA) from the catchment areas of the 10 health centers with probability proportional to size. Within each sampled EA, all households were screened in order to identify eligible women based on the criteria of delivering a live born child within 1 to 7 months prior to the survey. A sample size of 215 women was calculated to detect a 20-percentage point increase in the proportion of recent mothers who received the antenatal and postnatal services from the HEWs; to allow for up to 10% refusals, we targeted enrolling 240 women, or eight women per cluster. If more than eight eligible women were present in a cluster, the women were randomly chosen using a random number table. In six EAs fewer than eight women were found to be eligible, and other EAs were oversampled accordingly.

### Data collection

A standard questionnaire developed by the Saving Newborn Lives Program was adapted for this survey (see Additional file 1). The questionnaire includes modules on respondent and household characteristics, antenatal care, birth preparedness, delivery and immediate newborn care, nutrition, postnatal care for mother and baby, neonatal illness and care seeking and has been field tested and used in previous studies in Ethiopia by Save the Children. Data were collected between January 4 and 27, 2012, by six teams of two to four interviewers and one supervisor. All personnel were skilled data collectors with previous experience on Demographic and Health Surveys. Prior to the start of data collection, a five-day training was provided to the data collectors and supervisors to orient the teams to the study objectives and ensure that they had mastered the research protocol and instrument. Following the household screening and selection procedures, interviewers visited each selected woman at her home to administer the survey. If a selected woman was not at home on the first attempt to visit her, two additional attempts were made before another participant was selected in her place. Informed consent was obtained from each household for screening and from each sampled woman before proceeding with the survey questions.

### Data entry and analysis

Completed questionnaires were collected by supervisors in the field and transported to Addis Ababa for data entry. Double data entry was completed using a Microsoft Access database created for this survey. Two separate data clerks entered each form into a separate Access file. Discrepancies were identified and reconciled through reference to the original survey form. Additional data entry inconsistencies found during data exploration and analysis were recorded in an analysis log and corrected, when possible, by going back to the survey forms.

Of the original 224 cases included in the data set, six were excluded from analysis. Three were excluded because the case was a twin and the survey had already been completed for the first-born twin. In three other cases, the child was less than 28 days old at the time of the survey and therefore was not eligible according to the predetermined criteria. A total of 218 cases were included in the analysis. Key indicators were calculated for each of the survey modules using Stata 11 [26]. Stratified analyses by place of delivery were also calculated for newborn care indicators, and differences were tested for statistical significance using the chi-squared test. Sampling weights were calculated for clusters as the inverse of the proportion of eligible RDWs in that cluster selected for the survey, to account for the lower than expected sample in some clusters and oversampling in others. Confidence intervals and statistical tests were conducted using robust standard errors to adjust for survey design [27].

### Ethical approval

This study was approved by the Institutional Review Boards at the Johns Hopkins Bloomberg School of Public Health (IRB No. 3542) and the Ethiopia Health and Nutrition Research Institute (SERO 72-2-2011).

## Results

### Description of the sample

Among the 218 recently delivered women (RDWs) in the sample, 42.7% are from Amharra region, 28.9% from SNNP, 21.1% from Oromia, and 7.3% from Tigray (Table 2). The largest proportion of respondents were between the ages of 20 and 29 (57.8%), with an additional 34.9% of the sample between the ages of 30 and 39, and small proportions under 20 years (4.6%) or over 40 years old (2.8%). The vast majority of respondents were married (91.7%). The education levels reported by respondents were mixed, with 39.5% of respondents reporting no education and 11.4% reporting more than 10 years of education. The reported religion of RDWs is similar to the national breakdown, with 42.7% Orthodox Christian, 33.9% Muslim, and 22.9% Protestant Christian.

**Table 2:** Distribution of sample by background characteristics

**Characteristic**	**Frequency**	**Percentage**
Region		
Amharra	93/218	42.7%
Oromia	46/218	21.1%
SNNP	63/218	28.9%
Tigray	16/218	7.3%
Age of child		
4 to 10 weeks	55/218	25.2%
11 to 20 weeks	88/218	40.4%
21 to 31 weeks	75/218	34.4%
Sex of child		
Male	101/217	46.5%
Female	116/217	53.5%
Status of the child		
Alive	214/217	98.6%
Dead	3/217	1.4%
Age of respondent (mother)		
15 to 19	10/218	4.6%
20 to 29	126/218	57.8%
30 to 39	76/218	34.9%
40 or older	6/218	2.8%
Marital status		
Married	200/218	91.7%
Formerly married	13/218	6.0%
Never married	5/218	2.3%
Education		
None	83/210	39.5%
Grade 1 to 4	38/210	18.1%
Grade 5 to 8	43/210	20.5%
Grade 9 to 10	22/210	10.5%
Higher than grade 10	24/210	11.4%
Religion		
Orthodox	93/218	42.7%
Protestant	50/218	22.9%
Muslim	74/218	33.9%
Other	1/218	0.5%
Ethnicity		
Hadiya	30/218	13.8%
Oromo	33/218	15.1%
Amhara	96/218	44.0%
Tigre	18/218	8.3%
Gamo	23/218	10.6%
Wolayita	12/218	5.5%
Other	6/218	2.8%
Source of drinking water		
Piped water	125/218	57.3%
Well	15/218	6.9%
Spring water	70/218	32.1%
Surface water	8/218	3.7%
Type of toilet		
Ventilated improved latrine	13/218	6.0%
Pit latrine with slab	40/218	18.4%
Pit latrine with wood floor	63/218	28.9%
Open pit	53/218	24.3%
No facility/bush	49/218	22.5%
Asset ownership		
Radio	119/218	54.6%
Television	57/217	26.3%
Landline phone	13/218	6.0%
Mobile phone	124/218	56.9%
Bicycle	99/218	45.6%
Watch	79/218	36.2%

### Coverage of maternal and newborn health services

Over 80% of respondents reported making at least one antenatal care visit to a health facility, and 43.1% reported 4 or more visits (Table 3). Less than one-quarter of respondents (23.5%) initiated antenatal care before 16 weeks of pregnancy, as recommended. Most women reported receiving some antenatal care services from a nurse or midwife (72%), while 21% received antenatal care services from a HEW, and 19% reported being seen by a doctor (multiple responses allowed; data not shown). Among women attending antenatal care from any provider, the most frequently received counseling messages about newborn care were on breastfeeding (50%). Fewer women reported receiving counseling on newborn danger signs (19.6%), care of the low birth weight baby (LBW) (13.9%), and KMC positioning (8.1%).

**Table 3:** Utilization and receipt of maternal and newborn health services

**Indicator**	**Frequency**	**Weighted percentage**
		**(Adjusted CI)**
*Antenatal care*		
Proportion of RDWs who reported 1 or more ANC visits	184/217	82.7%
		(77.3, 88.2)
Proportion of RDWs who reported 4 or more ANC visits	103/216	43.1%
		(33.8, 52.4)
Proportion of RDWs who started ANC before 16 weeks	51/132	23.5%
		(14.2, 32.8)
Among women attending ANC, proportion receiving newborn care counseling		
Breastfeeding counseling	92/184	50.0%
		(39.6, 55.2)
Counseling on newborn danger signs	39/184	19.6%
		(13.1, 26.1)
Counseling on care of LBW baby	29/184	13.9%
		(8.1, 19.6)
Counseling on KMC positioning	16/184	8.1%
		(4.1, 12.1)
*Delivery care*		
Proportion of RDWs delivering at a health facility	78/218	28.8%
		(17.1, 40.4)
Attendant at delivery*		
Health worker	85/218	31.6%
		(19.3, 43.9)
Traditional birth attendant	63/218	31.7%
		(18.4, 45.0)
Relative/friend	79/218	40.1%
		(29.3, 50.8)
Other	31/218	16.6%
		(8.7, 24.6)
*Postnatal care*		
Proportion of RDWs who report a postnatal check by any health worker or volunteer community health worker in first week	27/218	10.6%
		(5.3, 15.9)

*More than one response possible.

The majority of women delivered their most recent child at home, with only 28.8% of women delivering in a health facility. The most common birth attendant that women reported was a relative or friend (40.1%), while equal proportions of women were attended by traditional birth attendants (31.7%) and health workers (31.6%), most notably a nurse midwife (27%), doctor (9%), or HEW (4%) (data not shown). Few women reported receiving a postnatal check by a health worker or volunteer in the first week after delivery (10.6%), whether at home or at a health facility.

### Newborn thermal care

Table 4 presents immediate newborn care practices as reported by women. Mothers reported that newborns were dried and/or wiped before delivery of the placenta for 63.2% of births, while they were wrapped for 82.3% of births. The most common immediate placements of the baby for home births were beside the mother (48.7%) or with someone else (15.9%), compared with a newborn bed/table (38.3%) or on the mother’s chest/belly (21.5%) for facility deliveries. In 7.7% of home births and 25.8% of facility births, the newborn was placed in skin-to-skin position at some point following the delivery. In only 25.3% of births did the mother report that bathing of the newborn was delayed at least 24 hours. Comparing facility and home births, drying and wrapping before delivery of the placenta, skin-to-skin position, and delayed bathing indicators were higher for facility deliveries, although these differences were not statistically significantly different. However, placing the baby on the mother’s chest immediately after delivery was significantly higher for facility deliveries (21.5%; CI: 9.9, 33.1) than home deliveries (2.1%; CI: 0, 4.6).

**Table 4:** Immediate newborn care in facility births vs home births

**Indicator**	**Overall**		**Home birth**		**Facility birth**		**p-value**
	**Frequency**	**Weighted percentage**	**Frequency**	**Weighted percentage**	**Frequency**	**Weighted percentage**	
		**(95% CI)**		**(95% CI)**		**(95% CI)**	
***Thermal care***							
Proportion of newborns who were wiped/dried before delivery of the placenta*	132/197	63.2%	84/136	58.9%	48/61	76.6%	0.0124
		(53.1, 73.3)		(48.5, 69.3)		(63.8, 89.4)	
Proportion of newborns who were wrapped before delivery of the placenta*	176/206	82.3%	114/137	80.5%	62/69	87.3%	0.2004
		(72.5, 92.1)		(69.8, 91.2)		(76.8, 97.8)	
Placement of newborn immediately after delivery							
On the floor	6/217	2.8%	6/140	3.4%	0/77	0%	0.2273
		(0.1, 5.5)		(0.1, 7.7)			
On the mother’s chest/belly	20/217	7.7%	3/140	2.1%	17/77	21.5%	0.000
		(2.6, 12.7)		(0, 4.6)		(9.9, 33.1)	
Beside the mother	72/217	38.5%	64/140	48.7%	8/77	12.9%	0.001
		(29.3, 47.8)		(40.4, 57.1)		(2.8, 23.1)	
With someone else	50/217	21.9%	43/140	27.0%	7/77	9.2%	0.0090
		(14.8, 29.0)		(16.9, 37.2)		(2.3, 16.1)	
On newborn bed/table	37/217	12.6%	5/140	2.3%	32/77	38.3%	0.000
		(5.3, 19.8)		(0, 5.2)		(25.2, 51.4)	
Other	24/217	13.0%	19/140	15.9%	5/77	5.7%	0.0240
		(6.4, 19.6)		(7.6, 24.3)		(1.2, 10.2)	
Don’t know	8/217	3.5%	0/140	0%	8/77	12.3%	0.0016
		(0.2, 6.9)				(14.0, 23.3)	
Proportion of newborns placed in skin-to-skin position at some point on the day of birth	29/216	12.9%	10/140	7.7%	19/76	25.8%	0.0036
		(6.7, 19.0)		(1.8, 13.6)		(13.6, 38.1)	
Proportion of newborns whose bathing was delayed at least 24 hours	59/214	25.3%	29/139	18.7%	30/75	42.3%	0.0071
		(16.7, 33.9)		(10.1, 27.4)		(27.1, 57.5)	
***Cord care***							
Article used to tie the cord							
New string/thread	106/217	45.8%	65/140	41.8%	41/77	55.8%	0.2060
		(30.2, 61.4)		(22.6, 61.0)		(41.1, 70.5)	
String/thread	22/217	8.3%	10/140	6.1%	12/77	13.8%	0.1057
		(3.8, 12.7)		(1.8, 10.4)		(3.9, 23.6)	
Fiber from ensete plant	34/217	22.3%	34/140	31.3%	0/77	0%	0.0102
		(6.7, 38.0)		(12.2, 50.4)			
Cord not tied	12/217	5.8%	12/140	8.1%	0/77	0%	0.2670
		(0, 12.3)		(0, 17.2)			
Other	44/217	27.8%	43/140	37.9%	1/77	2.6%	0.0010
		(11.3, 44.3)		(17.7, 58.0)		(0, 7.8)	
Don’t know	33/217	12.4%	10/140	6.2%	23/77	27.8%	0.0004
		(6.8, 17.9)		(1.3, 11.1)		(16.8, 38.9)	
Instruments used to cut the cord							
New razor blade	124/218	63.5%	122/140	88.3%	2/78	2.3%	0.0000
		(52.4, 74.6)		(83.4, 93.2)		(0, 5.7)	
Previously used razor blade	13/218	4.7%	12/140	6.2%	1/78	1.1%	0.0747
		(1.1, 8.3)		(1.0, 11.3)		(0, 3.3)	
Scissors	54/218	20.0%	2/140	1.4%	52/78	65.8%	0.0000
		(11.2, 28.7)		(0, 3.6)		(54.0, 77.6)	
Other	2/218	1.6%	2/140	2.3%	0/78	0%	0.2778
				(0, 5.2)			
		(0, 3.8)					
Don’t know	25/218	10.2%	2/140	1.8%	23/78	30.8%	0.0000
		(5.5, 14.8)		(0, 4.3)		(19.3, 42.2)	
Applications to the cord immediately after cutting							
Nothing applied	137/217	65.2%	99/139	72.6%	38/78	47.1%	0.0286
						(32.4, 61.8)	
		(54.3, 76.1)		(58.4, 86.8)			
Butter applied	36/217	16.9%	31/139	21.0%	5/78	7.0%	0.0208
		(8.2, 25.7)		(9.8, 32.1)		(0, 14.0)	
Other substance applied	8/217	3.0%	3/139	1.9%	5/78	5.7%	0.2367
		(0, 6.0)		(0, 4.6)		(0, 13.3)	
Don’t know	36/217	14.9%	6/139	4.6%	30/78	40.3%	0.0000
		(9.7, 20.0)		(1.3, 7.8)		(27.4, 53.2)	
***Nutrition***							
Proportion of newborns breastfed within the first hour	113/218	52.1%	69/140	50.2%	44/78	56.7%	0.3977
		(43.3, 60.8)		(38.8, 61.6)		(46.2, 67.2)	
Proportion of mothers who squeezed out and threw away the colostrum/first milk	94/217	44.5%	70/139	50.2%	24/78	30.4%	0.0160
		(34.2, 54.8)		(38.0, 62.5)		(18.9, 41.8)	
Proportion of newborns given something other than breast milk during the first 2 days	30/217	12.4%	18/139	11.9%	12/78	13.8%	0.6908
		(7.6, 17.2)		(5.8, 17.9)		(6.3, 21.1)	
Among newborns who were fed other foods/liquids during the first week, type of food given:**							
Plain water	8/30	32.7%	7/18	44.2%	1/12	8.2%	0.0571
		(11.1, 54.3)		(18.8, 69.5)		(0, 26.0)	
Sugar water	8/30	25.1%	4/18	21.4%	4/12	33.0%	0.5369
		(8.4, 41.8)		(9.4, 41.8)		(1.2, 64.7)	
Fresh butter	4/30	14.2%	4/18	20.9	0/12	0%	0.1308
		(0, 29.5)		(0.9, 9.5)			
Milk (other than breast milk)	5/30	13.2%	1/18	3.9%	4/12	33.0%	0.0265
		(1.3, 25.1)		(0, 12.1)		(4.9, 61.2)	
Other	11/30	41.6%	7/18	45.1%	4/12	34.0%	0.5325
		(19.5, 63.6)		(14.6, 75.6)		(14.3, 53.8)	

*“Don’t know” responses excluded; **More than one response allowed.

### Cord care

A new string or thread was used to tie the cord for 45.8% of births (Table 4), although the use of a string-like fiber from the ensete plant (known as the “false banana”) was also a common cord tie for home births (31.3% of home births), as were other methods of tying (37.9%). In home births the cord was most commonly cut with a new razor or blade (88.3%) or a previously used razor (6.2%), while scissors were most commonly used for facility deliveries (65.8%). Although 72.6% of women delivering at home reported that nothing was applied to the newborn’s cord after cutting, 21.0% reported that butter was applied to the area. Women who delivered at a facility most commonly reported that nothing was applied to the cord area (47.1%) or that they did not know whether any substance was applied (40.3%). The proportion of women reporting that they did not know how the cord was cut, tied, or whether anything was applied, was significantly higher for facility deliveries than home deliveries.

### Breast feeding

Only 52.1% of mothers reported that their newborns were breastfed within the first hour after delivery, with similar proportions for both home (50.2%) and facility (56.7%) deliveries (Table 4). Additionally, 44.5% of mothers reported that they squeezed out the colostrum before breastfeeding the newborn; this practice was less common for facility births (30.4%) compared to home births (50.2%), although differences were not statistically significant. A smaller proportion of mothers (12.4%) reported feeding their newborns food or liquid other than breast milk in the first two days. Among those newborns that were given other foods, the most commonly reported by mothers were plain water (32.7%), sugar water (25.1%), fresh butter (14.2%), and milk other than breast milk (13.2%).

### Knowledge of newborn danger signs

Mother’s unprompted knowledge of newborn danger signs was rather low, with only 29.3% of respondents able to name 3 or more danger signs out of a list of 11 (Table 5). The only newborn danger sign for which there was high awareness among mothers was fever (83.6%). To a lesser extent, mothers were also aware of poor feeding/suckling (39.5%), difficult/fast breathing (21.1%), lack of consciousness (17.3%), convulsions (12.7%), and red eyes (10.3%) as signs of serious newborn illness. Very few mothers listed other newborn danger signs, including cold temperature (8.5%), lethargy (3.5%), redness or discharge at the cord (1.7%), and yellow palms, eyes, or soles (0.4%).

**Table 5:** Knowledge of newborn danger signs, reported illness, and care seeking

**Indicator**	**Frequency**	**Weighted percentage**
		**(Adjusted CI)**
*Knowledge about danger signs*		
Proportion of mothers who can name at least 3 newborn danger signs (out of 11 signs)	66/218	29.3%
		(23.5, 35.0)
Proportion of mother listing specific danger signs unprompted		
Convulsions	28/218	12.7%
		(7.4, 18.0)
Fever	189/218	83.6%
		(76.2, 91.0)
Poor feeding/suckling	86/218	39.5%
		(30.8, 48.3)
Difficult/fast breathing	47/218	21.1%
		(16.4, 25.7)
Baby feels cold	20/218	8.5%
		(4.6, 12.3)
Baby too small/born too early	3/218	2.0%
		(0, 4.9)
Redness/discharge at cord	5/218	1.7%
		(0, 3.5)
Eyes red/swollen/discharge	21/218	10.3%
		(5.0, 15.6)
Yellow palms/soles/eyes	1/218	0.4%
		(0, 1.1)
Lethargy	8/218	3.5%
		(0.5, 6.5)
Unconscious	37/218	17.3%
		(11.4, 23.3)
**Reported newborn illness**		
Proportion of newborns reported to experience an illness	36/217	15.2%
		(8.7, 21.7)
Reported problems (from prompted list)^1^		
Fever	4/36	12.1%
		(0, 26.3)
Unable to suckle/feed	7/36	22.0%
		(7.9, 36.2)
Difficult/fast breathing	7/36	21.8%
		(7.4, 36.2)
Diarrhea	4/36	8.6%
		(0, 17.3)
Convulsions	3/36	7.0%
		(0, 15.6)
Persistent vomiting	10/36	30.6%
		(12.8, 48.4)
Yellow palms/soles/eyes	3/36	7.0%
		(0, 15.9)
Lethargy	4/36	9.1%
		(0, 19.4)
Unconscious	1/36	2.4%
		(0, 7.7)
Red/discharging eyes	2/36	4.3%
		(0, 10.4)
Skin pustules	2/36	4.8%
		(0, 12.5)
Redness or puss around the cord	0/36	0%
Other	15/35	45.3%
		(25.1, 65.6)
***Care seeking for newborn illness***		
Proportion of sick newborns taken to a government, private, or NGO health facility for treatment^2^	18/36	46.2%
		(21.1, 71.4)
Sources of care sought for sick newborns^1^		
Government health facility	15/36	38.1%
		(14.0, 62.3)
Private/NGO health facility	4/36	10.2%
		(0, 22.7)
Private pharmacy or other shop	4/36	11.8%
		(1.3, 22.3)
Traditional practitioner	1/36	3.5%
		(0, 9.9)
Among sick newborns who did not receive care outside the home, reason for not seeking care^1^		
Expecting self resolution of illness	10/14	70.2%
		(35.6, 100)
Health facility too far/no transport	5/14	41.7%
		(1.4, 82.0)
Not customary to seek care outside home	2/14	17.9%
		(0, 50.4)
Other	3/14	24.5%
		(0, 62.4)

^1^More than one response allowed; ^2^Private pharmacy excluded as health facility.

Thirty-six mothers (15.2%) reported that their babies experienced an illness during the newborn period. The most commonly reported illnesses from a prompted list included persistent vomiting (30.6%), inability to feed/suckle (22.0%), difficult/fast breathing (21.8%), and fever (12.1%). Among the 36 babies with newborn illness, 18 (46.2%) were taken to a health facility for treatment, including government hospitals, health centers or health posts (15 cases) and health facilities operated by private groups or nongovernmental organizations (4 cases). Mothers of 5 sick newborns reported seeking care at a private pharmacy or shop (4 cases) or a traditional healer (1 case). Mothers with sick newborns who did not seek care outside of the home (14 cases) reported that they expected the illness to resolve on its own (10 cases), that the health facility was too far (5 cases), or that it is not customary to seek care outside the home for illness (2 cases).

## Discussion

In this article we provide some of the first published statistics of newborn care practices in Ethiopia, for a representative sample of households within the catchment areas of 10 government health centers in four regions. This survey adds to a small but growing literature on newborn care practices at community level in Sub-Saharan Africa [18,28-32]. In the population served by the health facilities included in this study, the majority of women made one antenatal care visit to a health facility, but less than half made four or more visits. Women were most likely to deliver their babies at home, although facility delivery rates were higher among the study population than reported in the national Demographic and Health Survey (DHS) rural sample [17]. The population covered by this survey is slightly more urbanized than most rural areas in Ethiopia, and the indicators measured in this survey that are also measured by the DHS tend to fall in between the rates of the rural and urban DHS samples [17].

Although these results are not nationally representative, they do indicate areas where the newborn care practices of mothers and providers are consistent with WHO recommendations [7,33], and areas where improvements are needed. Common beneficial newborn care practices included exclusive breastfeeding, wrapping the baby before delivery of the placenta, and dry cord care. Practices contrary to WHO recommendations that were reported in this population of recent mothers include bathing during the first 24 hours of life, application of butter and other substances to the cord, and discarding of colostrum milk. We also report newborn care practices by place of delivery. The survey was not designed specifically to compare home births and facility births, so our sample sizes in each stratum are not large enough to detect smaller differences. However, point estimates suggest that there were not large differences for most essential newborn care indicators between facility and home deliveries, with the exception of delayed bathing and skin-to-skin care.

Improving newborn care and newborn health outcomes in Ethiopia will likely require a multifaceted approach. Increasing demand for and access to routine maternal and newborn health services at health facilities is an important challenge in Ethiopia, which has very low facility delivery rates in rural areas [17]. Ensuring high quality of care and counseling at health facilities is important for improving health outcomes and increasing demand for health services. Although this survey covered a limited set of provider-related newborn care practices, and is based on mothers’ recall rather than observations or interviews with service providers, the results suggest that providers may not always be following recommended newborn care practices or providing sufficient counseling for women on how to care for their newborns. The need for improvement in quality of maternal and newborn care is also highlighted by facility-based studies [34], and perceived low quality of care is reported as a reason that women in Ethiopia choose not to deliver at a health facility [35,36].

Services and interventions delivered at facility level only are not sufficient to meet the newborn health needs in the current context in Ethiopia. Given that the majority of births in Ethiopia take place at home, increased outreach and community programs are needed. The promotion of preventive newborn care practices through home visits by community health workers and community mobilization has been shown to reduce newborn deaths in high mortality settings in Asia [37]. Similar community education approaches for reducing under-five mortality were shown to be effective in Northern Ethiopia [38]. It has also been estimated that, in Ethiopia and Northern Nigeria, where facility delivery rates are low, high-impact newborn outreach interventions including oral antibiotics for severe newborn infections, could save 24,000 lives annually [39]. The results of this survey suggest that contacts between HEWs and pregnant women and mothers must increase for their counseling to reach a large population. About one-fifth of RDWs in this survey had contact with HEWs during ANC, but nurse/midwives were the most common providers, and few women had postnatal contact with any health provider. Based on these findings, the feasibility study is emphasizing increased home visits by HEWs, and utilization of the HDA 1-to-5 network, for promotion of recommended newborn care practices and KMC.

## Conclusions

Ethiopia has already made great initiatives to empower communities to improve maternal and child health through the HEW and HDA platforms. The Health Extension Program is credited with improving antenatal care utilization, use of family planning, and HIV testing during pregnancy [40]. The expansion of antenatal care through the HEWs, and the mobilization of community members through the HDA, can provide a strong basis to improve home-based practices through health education. HDA members are tasked with mobilizing the community and providing counseling on 64 key messages on maternal, newborn and child health issues. The work of the HEWs and HDAs have likely started to make a contribution to improving newborn care at community level, but baseline data on newborn care practices before the start of these programs are unfortunately not available. The incorporation of newborn care data into routine national surveys, such as the DHS and UNICEF’s Multiple Indicator Cluster Survey (MICS), is critical for identifying gaps in newborn health, targeting interventions, and monitoring progress [9].

## Abbreviations

ANC: Antenatal care; DHS: Demographic and Health Survey; FMOH: Federal Ministry of Health; HEW: Health Extension Workers; HDA: Health Development Army; KMC: Kangaroo mother care; RDW: Recently-delivered woman; SNNP: Southern Nations, Nationalities, and People Region; WHO: World Health Organization.

## Competing interests

The authors declare that they have no competing interests.

## Authors’ contributions

JJ, BR, AB, BW and JCK conceived of and designed the study. JCK, AS, ED, BW, and MT adapted the study instruments. JC and AB developed the data collection protocols and AS, ED, and BW supervised data collection. JCK performed the statistical analysis. JCK and MT wrote the first draft of the paper. All authors read and approved the final manuscript.

## Pre-publication history

The pre-publication history for this paper can be accessed here:

http://www.biomedcentral.com/1471-2431/13/198/prepub

## Supplementary Material

Additional file 1 **Questionnaire for women who had a delivery from 1 to 7 months ago.** Description: Study instrument used during data collection. Click here for file
