# Supplementary material for: Newborn care practices at home and in health facilities in 4 regions of Ethiopia
Source: BMC Pediatr. 2013 Dec 1;13:198. doi: 10.1186/1471-2431-13-198 (PMC4219496; doi:10.1186/1471-2431-13-198)
Supplement: Additional file 1 — Questionnaire for women who had a delivery from 1 to 7 months ago. Description: Study instrument used during data collection. [file 1471-2431-13-198-S1.docx]

**MCHIP, Ethiopia—Baseline Survey**

**Questionnaire for women who had a delivery from 1 to 7 months or 30-209 days ago**

| **Identification** | **Code** |
| --- | --- |
| **REGION**  **SNNPR…..…1**  **Oromia……2**  **Tigray…….3**  **Amhara…..4** | **HEALTH CENTER**  **Code [_____\|_____]** |
| **KEBELE**  **Name________________ Code [_____]** | **GASHA/GERE**  **Name________________** |
| **HOUSEHOLD NUMBER**  **[_______\|________\|_______]** |  |
|  | **Child’s Birth Date**  **________/__________/______________**  **Day Month Year** |
| **Is child alive or deceased? (1=Alive, 2=Deceased)** |  |
| **Child’s Age**  **If alive, record child’s current age. If deceased, record age at death.**  **If less than 30 days, record baby’s age in days Days** [_____ \| _____]  **If greater than 30 days record in weeks**  **Weeks** [_____ \| _____] | |
| **Sex of Child (1=Male, 2=Female)** |  |

| **Section 1. Respondent and Household Characteristics** | | | | |
| --- | --- | --- | --- | --- |
| T1 | **Time at beginning of Interview** | | **_______:_______** | |
| *Now I would like to ask you some questions about you and your household.* | | | | |
| 101 | | In what month and year were you born? | Month [_____ \| _____]  Don’t know month………………..99  Year [_____ \| _____\|_____\|_____]  Don’t know year………………..9999 | SKIP |
| 102 | | How old are you?  ***Compare and correct 101 and/or 102 if inconsistent. Probe using local calendar/historical events.*** | Age in completed years [_____ \| ______]  Don’t know………………………..99 |  |
| 103 | | What is your current marital status? | Married/Living together..…………...…………. ….….…. 1  Formerly Married (divorced, widowed, separated)………..2  Never Married…………………………………. ….….…. 3 |  |
| 104 | | Have you ever attended school? | Yes . . . . . . . . . . . . . . . . . . . . . . . . . . . . . . 1  No . . . . . . . . . . . . . . . . . . . . . . . . . . . . . . . 2 | 106 |
| 105 | | What is the highest class you completed | Grade 1 to 4…………………….. .1  Grade 5 to 8……………………….2  Grade 9 to 10 ……………………. 3  Higher than Grade 10 …………… 4 |  |
| 106 | | Now I would like you to read out as much of this sentence as you can.  ***Show card to the respondent*** | Cannot read at all…………………….1  Able to read parts of sentence………...2  Able to read whole sentence………….3 |  |
| 107 | | What is your religion? | Orthodox……………………………..1  Catholic………………………………2  Protestant…………………………….3  Muslim………………………………4  Other…………………………………5  Specify________________________________ |  |
| 108 | | What is your ethnicity?  ***Record the major ethnic group*** | Hadiya……………………………..1  Oromo..……………………………2  Amhara…………………………….3  Tigre……………………………….4  Gamo………………………………5  Wolayita……………………………6  Other………………………………7  Specify________________________________ |  |
| 109 | | Main material of the roof of the house  ***Record observation*** | Natural roofing  Thatch/Leaf......................................1  Rudimentary roof  Tin....................................................2  Rustic mat/plastic sheets…………..3  Reed/Bamboo………………………4  Wood planks……………………….5  Finished roofing  Corrugated iron ……………………6  Wood ………………………………7  Other………….……………………8  Specify __________________________ |  |

| 110 | Main material of the wall  ***Record observation*** | Natural walls  No walls . . . . . . . . . . . . … . . 1  Cane/trunks/bamboo/reed ..… 2  Rudimentary walls  Bamboo/wood with mud . . . .3  Stone with mud . . . . . .... . . .4  Finished walls  Cement . . . . . . . . . . . . . . .….5  Stone with lime/cement . . ….6  Wood planks/shingles . . …… 7  Other ………………………………… 8  Specify______________________________ |  |
| --- | --- | --- | --- |
| 111 | Main material of the floor  ***Record observation*** | Natural floor  Earth/Sand…………………………...1  Dung………………………………....2  Rudimentary floor  Wood flanks........................................3  Reed/ Bamboo ...................................4  Finished floor  Cement/concrete/bricks......................5  Polished wood……………………....6  Other …………………………………7  Specify______________________________ |  |
| 112 | What is the main source of drinking water for members of your household? | PIPED WATER  Piped into dwelling . . . . . . . . 1  Piped into compound . . . . . .2  Piped outside compound ……3  TUBE WELL OR BOREHOLE . . . . . .4  DUG WELL  Protected well . . . . . . . . . . …5  Unprotected well . . . . . . . …. 6  WATER FROM SPRING  Protected spring . . . . . . . ….. 7  Unprotected spring . . . . . …..8  RAINWATER . . . . . . . . . . . . . . . . . .. . 9  TANKER TRUCK . . . . . . . . . . . . . . . .10  SURFACE WATER  River/dam/Lake/pond/stream/canal/ Irrigation channel) ……………………11  OTHER…………………………………12  Specify_____________________________ |  |
| 113 | What kind of toilet facility do members of your household usually use?  ***Record observation*** | LATRINE  Ventilated improved pit latrine (VIP). . . . . 1  Pit latrine with slab . . . . . ……………….. 2  Pit latrine with wood floor………………..3  Open pit . ……………………….. . . . . . . .4  NO FACILITY/BUSH/FIELD . . . . . . …..5  OTHER…………………………………..6  Specify______________________________ | 115 |
| 114 | Do you share this toilet facility with other households? | Yes . . . . . . . . . . . . . . . . . . . . . . . . . . . . . . 1  No . . . . . . . . . . . . . . . . . . . . . . . . . . . . . . 2 |  |
| 115 | Does your household have:  Electricity?  A radio?  A television?  A non-mobile telephone?  A mobile telephone?  Watch?  A table?  A chair?  Bed  A mattress?  A Bicycle? | \|  \| Yes \| No \| \| --- \| --- \| --- \| \| 1. Electricity……………………….. \| 1 \| 2 \| \| 1. Radio …………………………….. \| 1 \| 2 \| \| 1. Television ……………………….. \| 1 \| 2 \| \| 1. Non-mobile telephone …………… \| 1 \| 2 \| \| 1. Mobile telephone ……………….. \| 1 \| 2 \| \| 1. Watch …………………………….. \| 1 \| 2 \| \| 1. Table …………………………….. \| 1 \| 2 \| \| 1. Chair……………………………… \| 1 \| 2 \| \| 1. Bed…………………………. \| 1 \| 2 \| \| 1. Mattress…………………………….. \| 1 \| 2 \| \| 1. Bicycle…………………………….. \| 1 \| 2 \| |  |
| 116 | How long does it take you to walk to the nearest health center?  What about the health post?  ***If less than an hour, record it in minutes, and write 00 in space for hour.*** | HEALTH CENTER  Minutes [____\|____]  Hours [____\|____]  HEALTH POST  Minutes [____\|____]  Hours [____\|____] |  |

| **Section 2. Antenatal Care**  *Now I would like to ask you some questions about services you may have received during your pregnancy* | | | |
| --- | --- | --- | --- |
| 201 | Did you see anyone for antenatal care for your last pregnancy? | \| Yes…………………… \| 1 \| \| --- \| --- \| \| No…………………… \| 2 \| | SKIP  301 |
| 202 | IF YES: Whom did you see?  Anyone else?  ***Probe to identify each type of person and record all mentioned.*** | Mentioned: YesNo  HEALTH PERSONNEL   1. Doctor . . . . . . . . …..……………..1 2 2. Nurse/Midwife….. …………………....1 2 3. Health extension worker………………1 2 4. Health worker unknown type….…….,,.1 2   OTHER PERSON   1. Traditional Birth Attendant………..…….1 2 2. Other…………………………………….1 2   Specify ___________________________________________ |  |
| 203 | Where did you receive antenatal care for this pregnancy?Anywhere else?  ***Probe to identify type(s) of source(s) and circle the appropriate code(s). Circle all mentioned.*** | Mentioned: YesNo  HOME   1. Your home . . . …………………….….1 2 2. Other home . . . ……………….………1 2   PUBLIC SECTOR   1. Govt. Hospital. . . . ...............................1 2 2. Govt. Health center . . . . . ...................1 2 3. Govt. Health post…… ........................ 1 2 4. NGO Clinic…………………………....1 2 5. Other….………………..…………….1 2   Specify______________________________________ |  |
| 204 | How many months pregnant were you when you first received antenatal care for this pregnancy? | Months  Do not know…………99 |  |
| 205 | How many times did you receive antenatal care during this pregnancy? | Number of times  Do not know…………99 |  |
| 206 | Now I would like to ask you about the antenatal care you received specifically from Health Extensions Workers, either at home or at the health post.  During your pregnancy, did a Health Extension Worker, do the following:   1. Were you weighed? 2. Was your blood pressure measured? 3. Counseled on nutrition during pregnancy? 4. Counseled on birth preparedness? 5. Counseled on breastfeeding? 6. Counseled on HIV? 7. Counseled on care of Low Birth Weight Baby? 8. Counseled on Family Planning? 9. Coached on how to put baby in KMC position? 10. Counseled on expressing breast milk? 11. Counseled on cup feeding with Breast milk? 12. Counseled on newborn danger signs 13. Other | \|  \| Yes \| No Don’t Know \| \| --- \| --- \| --- \| \| 1. Weight ……. \| 1 \| 2 9 \| \| 1. BP………… \| 1 \| 2 9 \| \| 1. Nutrition…… \| 1 \| 2 9 \| \| 1. Birth preparedness \| 1 \| 2 9 \| \| 1. Breastfeeding \| 1 \| 2 9 \| \| 1. HIV \| 1 \| 2 9 \| \| 1. Low birth weight \| 1 \| 2 9 \| \| 1. Family Planning \| 1 \| 2 9 \| \| i) KMC position \| 1 \| 2 9 \| \|  \|  \|  \| \| j) Express breast milk \| 1 \| 2 9 \| \| k) Cup feeding \| 1 \| 2 9 \| \|  \|  \|  \| \| l) newborn danger signs \| 1 \| 2 9 \| \| m) Other  Specify ___________ \| 1 \| 2 9 \| |  |
| 207 | Now I would like to ask you about the antenatal care you received from other health workers, not the HEW.  During your pregnancy, did any other health worker at the health center or hospital, do the following:   1. Were you weighed? 2. Was your blood pressure measured? 3. Counseled on nutrition during pregnancy? 4. Counseled on birth preparedness? 5. Counseled on breastfeeding? 6. Counseled on HIV? 7. Counseled on care of Low Birth Weight Baby? 8. Counseled on Family Planning? 9. Coached on how to put baby in KMC position? 10. Counseled on expressing breast milk? 11. Counseled on cup feeding with Breast milk? 12. Counseled on newborn danger signs 13. Other | \|  \| Yes \| No Don’t Know \| \| --- \| --- \| --- \| \| 1. Weight ……. \| 1 \| 2 9 \| \| 1. BP………… \| 1 \| 2 9 \| \| 1. Nutrition…… \| 1 \| 2 9 \| \| 1. Birth preparedness \| 1 \| 2 9 \| \| 1. Breastfeeding \| 1 \| 2 9 \| \| 1. HIV \| 1 \| 2 9 \| \| 1. Low birth weight \| 1 \| 2 9 \| \| 1. Family Planning \| 1 \| 2 9 \| \| i) KMC position \| 1 \| 2 9 \| \|  \|  \|  \| \| j) Express breast milk \| 1 \| 2 9 \| \| k) Cup feeding \| 1 \| 2 9 \| \|  \|  \|  \| \| 1. Newborn danger signs 2. Other   Specify ________ \| 1 \| 2 9 \| |  |

| **Section 3. Birth Preparedness**  ***Now I would like to ask you some questions about how you prepared for the arrival of your baby.*** | | | |
| --- | --- | --- | --- |
| **No.** | **Questions and Filters** | **Coding Categories** | **Skip** |
| 301 | During your last pregnancy, did you know your due date, or when you would expect to deliver the baby? | Yes…………………………….1  No……………………………..2 |  |
| 302 | During your last pregnancy did you make any preparations for your delivery?  ***Read each option and mark yes or no.*** | Mentioned: YesNo   1. Financial……………………………..1 2 2. Transport…………………………….1 2 3. Food………………………………….1 2 4. Identification of birth attendant……...1 2 5. Identification of facility……...………1 2 6. Materials for clean delivery………… 1 2 7. In case of emergency………………...1 2 8. Other…………………………………1 2   Specify________________________________________ |  |
| 303 | Did you or members of your family set aside any money specifically for care during the delivery? | Yes…………………………….1  No……………………………..2 | 306 |
| 304 | How much money did you set aside specifically for care during the delivery? | ETB ___________________  Don’t Know………………..999 |  |
| 305 | How did you/your family get the money for the delivery?  ***DO NOT READ OUT ANSWERS****.*  **(Circle all responses)** | Saved little by little……………….…1  Member of savings scheme……….…2  Borrowed……………………………3  Sold livestock………..........................4  Performed labor…………………… .5  Other… ……………………………..6  Specify___________________________  Don’t Know…………………. . . . . . 9 |  |
| 306 | Who did you plan to attend your delivery?  ***DO NOT READ OUT ANSWERS****.*  ***Probe: “Did you do plan anyone else to attend the delivery?”***  **(Circle all responses)** | Mentioned: YesNo   1. Health worker at facility ………………. . 1 2 2. Traditional Birth Attendant………………1 2 3. Mother……………………………………1 2 4. Mother-in-law……………………………1 2 5. Other female relative…………….……….1 2 6. Health Extension Worker………...............1 2 7. Community health volunteer………….…1 2 8. Other..………………………………...….1 2   Specify______________________   1. No one…………………………………...1 2 |  |
| 307 | During your last delivery did you plan for a place to deliver your child? | Yes…………………………….1  No……………………………..2 | 401 |
| 308 | Where did you plan to deliver your child? | HOME  Your home. . ……………………….. 1  Other home. . ………………………. 2  PUBLIC SECTOR  Govt. Hospital……………………….3  Govt. Health center . . . . . …………. 4  Govt. clinic………………………….5  Govt. Health post . . . . . . . ………. .6  NON-GOVT(NGO) HEALTH FACILITY……7  PRIVATE MED. SECTOR  Pvt. Hospital/clinic.……………..…. 8  OTHER ……………………………………….9  Specify__________________­­­­_____________ |  |
| **Section 4. Delivery and Immediate Newborn Care**  ***Now I would like to ask you some questions about the delivery of your baby.*** | | | |
| **No.** | **Questions and Filters** | **Coding categories** | **Skip** |
| 401 | Where did you give birth to (NAME)?  Probe to identify the type of source and circle the appropriate code.  If unable to determine if a hospital, health center, or clinic is public or private medical, write the name of the place.  ____________________________________  (NAME OF PLACE) | HOME  Your home. . ……………………….. 1  Other home. . ………………………. 2  PUBLIC SECTOR  Govt. Hospital……………………….3  Govt. Health center . . . . . …………. 4  Govt. clinic………………………….5  Govt. Health post . . . . . . . ………. .6  NON-GOVT(NGO) HEALTH FACILITY……7  PRIVATE MED. SECTOR  Pvt. Hospital/clinic.……………..…. 8  OTHER ……………………………………….9  Specify__________________­­­­_____________ | 403  403  403  403  403  403 |
| 402 | What was the reason you didn’t deliver in a health facility?  Do Not Read Out Responses  PROBE: Any other reason?  **(Record all mentioned).** | Mentioned: YesNo   1. Preferred to deliver at home………… .1 2 2. Cost too much. ……………… ………1 2 3. Too far/ no transportation…………… .1 2 4. Not necessary …………………………1 2 5. Not customary…………………………1 2 6. Delivered on the way to health facility…………………………1 2 7. Other………………………………….1 2   Specify _____________________________________ |  |
| 403 | Who assisted with the delivery of (NAME)?  Anyone else?  Probe for the type(s) of person(s) and record all mentioned. If respondent says ‘no one assisted’, probe to determine whether any adults were present at the delivery. | Mentioned: YesNo  HEALTH PERSONNEL   1. Doctor ………………………..1 2 2. Nurse/midwife.…. …………… 1 2 3. Health extension worker……….1 2 4. Health worker unknown type….1 2   OTHER PERSON   1. Traditional birth attendant. …...1 2 2. Relative/friend…………………1 2 3. Other……………………………1 2   Specify__________________________________   1. NoOne . . . . …………………..1 2 | 405  405  405  405 |

| 404 | Was (NAME) delivered by caesarean section (surgery)? | Yes………………….1  No…………………..2 |  |
| --- | --- | --- | --- |
| ***Now I would like to ask you what happened immediately after delivery*** | | | |
| 405 | After birth, was the baby placed skin-to-skin on your belly/chest? | Yes………………….1  No…………………..2 | 407 |
| 406 | When was the baby placed skin-to-skin on your belly/chest?  ***Read out responses*** | Mentioned: YesNoDon’t Know  SKIN-TO-SKIN   1. Before cord cut…………………..….129 2. Before placenta expelled……………1 2 9 3. Before baby dried……………...........1 2 9 4. Other……………….………….……1 2 9   Specify _________________________________ |  |
| 407 | Was the baby wiped (dried) before the placenta was delivered? | Yes………………….1  No…………………..2  Don’t know . . . …….9 |  |
| 408 | Was the baby wrapped with cloth before the placenta was delivered? | Yes………………….1  No…………………..2  Don’t know . . . …….9 |  |
| 409 | Did your baby cry or breathe easily immediately after birth? | Yes………………….1  No…………………..2  Don’t know . . . …….9 | 412 |
| 410 | What was done to help the baby cry or breathe at the time of birth, if anything?  ***Please do not read out responses.***  ***ASK: Anything else?***  ***Record all responses.*** | Mentioned: YesNo   1. Rubbed/massaged………………1 2 2. Dried. ………………………….1 2 3. Mouth cleared …………………1 2 4. Nothing…………………………1 2 5. Other……………………………1 2   Specify _________________________________  Don’t know . . . . ………………9 | 412  412 |
| 411 | Who took these measures to help the baby cry or breathe? | HEALTH PERSONNEL  Doctor …………………………………..1  Nurse/midwife…. …………………….… 2  Health extension worker…………….……3  Health worker unknown type…………….4  OTHER PERSON  Traditional birth attendant. ………. …….5  Relative/friend……………………………6  Other……………………………………..7  (specify)_________________________________ |  |
| 412 | Where was the baby placed immediately after delivery? | On the floor …………….………….… 1  On the mother’s belly/chest..……..….. 2  Beside the mother……………………...3  With someone else …………………... 4  On newborn bed/table ………………...5  Other…………………………………...6  Other (specify) __________________________________  Don’t know ……………………….... ………9 |  |
| 413 | Was there a person who took care of the newborn while you were delivering the placenta? | Yes …........................................ ….................1  No …...............................................................2  Don’t Know/Can’t Remember….....................9 | 415  415 |
| 414 | Who took care of the newborn? | HEALTH PERSONNEL  Nurse/midwife…. ……………………… 1  Health extension worker…………………2  Health worker unknown type……………3  OTHER PERSON  Traditional birth attendant. ……….…….4  Mother/Mother-in-law…………………...5  Sister/sister-in-law………………………..6  Other female relative……………………..7  Other……………………………………...8  Other (specify)_________________________  NO ONE . .. . ……………………………............... 9 |  |
| 415 | What was used to tie the cord? | New string or thread……………………….....1  String, or thread …………….………..............2  Cord was not tied …………………………. .3  Other…………………………………....…….4  Other (specify) ____________________________________  Don’t know …………………………...9 | 417 |
| 416 | Was the tie/string used to tie the cord boiled prior to use? | Yes …........................................ …..................1  No …................................................................2  Don’t Know/Can’t Remember…......................9 |  |
| 417 | What was used to cut the cord? | New razor blade…............................................1  Razor blade …………………………………..2  Scissors……………………………………….3  Other.. …..........................................................4  Other (specify) __________________________________  Don’t Know/Can’t Remember…......................9 |  |
| 418 | Was the instrument used to cut cord boiled prior to use? | Yes …........................................ ….................1  No …...............................................................2  Don’t Know/Can’t Remember….....................9 |  |
| 419 | Was anything applied to the cord immediately after cutting (and tying)? | Yes ………………………………………….1  No………………………………………….. 2  Don’t Know..……………………………......9 | 421  421 |
| 420 | What was applied to the cord just after cutting the cord? | Butter…………………………….……….…1  Ash………………………………………….2  Ointment……….……………..…………..…3  Animal dung…………………...…………....4  Oil….……………………………………..…5  Cold water…………………………………..6  Other………………………………………...7  Other (specify) _______________________  Don’t know…………………….……………8 |  |
| 421 | How much did (NAME) weigh at birth?  ***BIRTH WEIGHT TAKEN WITHIN 7 DAYSOF DELIVERY. DO NOT RECORD IF BEYOND THE FIRST WEEK.***  ***RECORD WEIGHT FROM HEALTH***  ***CARD, IF AVAILABLE.*** | KG from card in kilograms _____.______  KG from recall______.______  Baby not weighed………………..96  Don’t know………. ……………..98 |  |
| 422 | When (NAME) was born, was he/she very large, larger than average, average, smaller than average, or very small? | Very large . . . . . . ……………………..1  Larger than average . . . . . …………… 2  Average . . . . . . . …………………….. 3  Smaller than average. …………………4  Very small . . . . . …………………….. 5  Don’t know . . . . …………………… . 9 | 430  430  430  430 |
|  | **For mothers who reported perceiving their babies to be small or reported a birth weight of less than 2.5K g** | |  |
| 423 | Since your baby was small do you know if your baby was born too early?  ***Probe: Before full term?*** | Yes……………………1  No…………………….2  Don’t know…………...9 |  |
| 424 | Since your baby was small, did you receive extra visits or care for your baby? (Eg. more visits than normal, referral to hospital) | Yes……………………1  No…………………….2  Don’t know…………...9 |  |
| 425 | Since your baby was small, what extra care did you give to your baby?  ***Please do not read out responses.***  ***ASK: Anything else?***  ***Record all responses.*** | Mentioned: YesNo   1. More frequent breastfeeding……….1 2 2. Skin-to-skin contact.…….. . . . . . …1 2 3. Nothing……… . . . . . . . ………….1 2 4. Other……………………………… 1 2   Specify ___________________________________  Don’t know . . . . ………………… . 9 |  |
| 426 | Did you put your baby in KMC position? | Yes………………….1  No…………………..2 (skip) | 430 |
| 427 | When did you first put your baby in KMC position? | Immediately after birth ……………………1  In the first 24 hours after delivery ……..…..2  In the first three days after delivery………..3  In the first week after delivery……………..4  After the first week of delivery …………. . 5 |  |
| 428 | How many days was (name) in KMC position, at least part of the day, until you no longer put (name) in KMC position? | ____________________days  Don’t remember-----------998 |  |
| 429 | During the days that (name) was in KMC position, how long did you keep him/her in KMC position? | The whole day (daytime and night time)……………………..…..1  About half a day (only daytime or only nighttime)……………....2  About quarter of a day (half daytime or half nighttime)…………3  Less than a quarter of a day…………………………………...…4  Don’t remember……………………………………………...…..8 |  |
| 430 | In the first two days of life was (name) breastfed or cup-fed? | Breastfed only…………………………………………..1  Cup fed with breast milk only…………………..............2  Cup fed with non-breast milk products only……………3  Both breastfed and cup fed with breastmilk………….....4  Both breastfed and cup fed with non-breastmilk………..5  Bottle fed with breast milk only ……………………..…6  Bottle fed with non-breast milk products only………….7  Other…………………………………………………….8  Don’t know………………………………………….…..9 |  |
| 431 | How often was (name) breastfed or cup fed?  Probe for number of times fed during the day and number of times fed during the night | ______________number of times |  |
| 432 | How long after birth was (NAME) bathed for the first time?  **If less than one day, probe to record the number of hours of life when bathed**    **If less than one hour, enter “00”** | Hours………………………….  Days.………………………….  Don’t Know………………..………….99 |  |
| 433 | Was the baby put to the breast before the placenta was delivered? | Yes………………….1  No…………………..2  Don’t know . . . …….9 |  |
| 434 | How long after birth did you first put (NAME) to the breast?  **If less than 1 hour, record ‘00' hours.**  **If less than 24 hours, record hours. Otherwise, record days.** | Hours………………………….[_____\|_____]  Days.…………………………. [_____\|_____]  Don’t Know………………..………….98  Never breastfed……………..…………99 |  |
| 435 | Did you squeeze out and throw away the first milk? | Yes…………………1  No………………...2 |  |
| 436 | In the first three days after delivery, was (NAME) given anything to drink other than breastmilk? | Yes…………………1  No………………...2 | 438 |
| 437 | What was (NAME) given to drink?  Anything else?  Record all liquids mentioned. | Mentioned: Yes No   \| 1. Milk (other than breast milk)……. 1 2 \| \| --- \| \| 1. Plain water………………………..1 2 \| \| 1. Sugar or glucose water……………1 2 \| \| 1. Fruit juice…………………………1 2 \| \| 1. Infant formula…………………….1 2 \| \| 1. Tea/infusions/”hamesa”………….. 1 2 \| \| 1. Fresh Butter……………………….1 2 \| \| 1. Fenugreek…………………………1 2 \| \| 1. Other………………………………1 2 \| \| Specify__________________________________ \| |  |
| 438 | What did you (or the birth attendant) do to keep (NAME) warm following delivery?  Anything else?  Multiple responses possible  (Circle all responses mentioned) | Mentioned: Yes No   1. Dried the baby ………………………….1 2 2. Wrapped the baby with clean cloths…….1 2 3. Put baby beside the mother……………...1 2 4. Kept the baby on bare skin to   skin contact…………1 2   1. Bathed in warm water ……………………1 2 2. warmed delivery room…………………...1 2 3. Other……………………………………..1 2   specify ____________________________________  Nothing done…………………………….…3  Don’t know………………………………....9 |  |

| **Section 5. Postnatal Care for Mother & Baby** | | | |
| --- | --- | --- | --- |
| 501 | After (NAME) was born, did any health care provider or volunteer community health worker check on your or your baby’s health in the first week?  PROBE: Any visits where the health care provider or CHW came to your home after delivery to talk, provide counseling, examine yourself or your baby? | Yes . . . . . . . . . . . . . . 1  No . . . . . . . . . . . . . . 2 | 504 |
| 502 | After (NAME) was born, did a Health Extension Worker visit your home to check on your baby’s health in the first week? | Yes . . . . . . . . . . . . . . 1  No . . . . . . . . . . . . . . 2 | 504 |
| 503 | How many visits did you and/or your baby have from a Health Extension Worker in the first week?  PROBE: Ask Separately for baby & mother, and for visits where both were seen.  PROBE: Any counseling given to the Mother for care of her baby is considered a visit for the baby | Mother Baby Both at same  only _________ only________ time_____ |  |
| 504 | BABY VISIT 1  How long after delivery did the HEW visited you at home to check on your baby’shealth?  If less than one day, record hours. If less than one week, record days. | \| Hours………………. \|  \|  \| \| --- \| --- \| --- \| \| Days……………….. \|  \|  \| \| Weeks……………… \|  \|  \| \| Don’t know…...........99 \|  \|  \| \| Never received visit from HEW………………..4 \|  \|  \| | 510 |
| 505 | BABY VISIT 1  What did the HEWdo during that visit to check the health of your baby? | Mentioned: YesNo   1. Generally examined/   looked at baby’s body…1 2   1. Weighed baby……………………1 2 2. Checked Cord………..…..……….1 2 3. Counseled on Breastfeeding………1 2 4. Observed Breastfeeding . . . . . . .1 2 5. Counseled on skin-to-skin contact/   warmth…..1 2  g) Observed KMC positioning ……12   1. Checked baby for danger signs …..1 2 2. Counseled on danger signs………..1 2 3. Referred to health center/hospital…1 2 4. Nothing……………………………1 2 5. Other………………………………1 2   Specify_____________________________________ |  |
|  | **IF MORE THAN ONE VISIT FOR THE BABY ASK 505-508. IF NO ADDITIONAL VISITS, SKIP TO 509** | |  |
| 506 | BABY 2  How long after delivery did the second check take place?  If less than one day, record hours. If less than one week, record days. | \| Hours……………… \|  \|  \| \| --- \| --- \| --- \| \| Days……………….. \|  \|  \| \| Weeks……………… \|  \|  \| \| Don’t know…...........99 \|  \|  \| \| Didn’t receive second visit from HEW……………..4 \|  \|  \| | 510 |
| 507 | BABY 2  What did the HEW do during that visit to check the health of your baby? | Mentioned: YesNo   1. Generally examined/   looked at baby’s body…1 2   1. Weighed baby……………………1 2 2. Checked Cord………..…..……….1 2 3. Counseled on Breastfeeding………1 2 4. Observed Breastfeeding . . . . . . .1 2 5. Counseled on skin-to-skin contact/   warmth…..1 2  h) Observed KMC positioning ……12   1. Checked baby for danger signs …..1 2 2. Counseled on danger signs………..1 2 3. Referred to health center/hospital…1 2 4. Nothing……………………………1 2 5. Other………………………………1 2   Specify_____________________________________ |  |
| 508 | BABY VISIT 3  How long after delivery did the third check take place? | \| Hours………………. \|  \|  \| \| --- \| --- \| --- \| \| Days……………….. \|  \|  \| \| Weeks……………… \|  \|  \| \| Don’t know…...........99 \|  \|  \| \| Didn’t receive 3^rd^ visit from HEW…………..4 \|  \|  \| | 510 |
| 509 | BABY VISIT 3  What did the HEW do during that visit to check the health of your baby? | Mentioned: YesNo   1. Generally examined/   looked at baby’s body…1 2   1. Weighed baby……………………1 2 2. Checked Cord………..…..……….1 2 3. Counseled on Breastfeeding………1 2 4. Observed Breastfeeding . . . . . . .1 2 5. Counseled on skin-to-skin contact/   warmth…..1 2  h) Observed KMC positioning ……12   1. Checked baby for danger signs …..1 2 2. Counseled on danger signs………..1 2 3. Referred to health center/hospital…1 2 4. Nothing……………………………1 2 5. Other………………………………1 2   Specify_____________________________________ |  |
| 510 | Sometimes newborns, within the first month of life, have severe illnesses and should be taken immediately to a health facility.  What types of symptoms would cause you to take your newborn to a health facility right away?  **Prompt: “Anything else?”**  **But do NOT prompt with any suggestions.**  **Circle all symptoms mentioned** | Mentioned: Yes No   1. Convulsions ………………………1 2 2. Fever ……………………………...1 2 3. Poor suckling or feeding……….…1 2 4. Child has difficult/fast breathing… 1 2 5. Baby feels cold …………………...1 2 6. Baby too small or born too early…1 2 7. Redness/Discharge around cord….1 2 8. Red swollen eyes/discharge………1 2 9. Yellow palms/soles/eyes….….1 2 10. Lethargy………………………….1 2 11. Unconscious……………………..1 2 12. Other……………………………..1 2   Specify_______________________________  Don’t Know……….……………8 |  |
|  |  |  |  |

| **Section 6. Neonatal Illness & Care Seeking**  *Now I would like to ask you questions about your baby’s state of health.* | | | |
| --- | --- | --- | --- |
| **No.** | **Questions and filters** | **Coding categories** | **Skip** |
| 601 | Did (Name) experience any health problems during the first month following delivery? | Yes……………………………………………1  No……………………………………………..2  Don’t know……………………………………8 | SECTION 7  SECTION 7 |
| 602 | What were the health problems?  **AT FIRST DO NOT PROPMT**  **PROBE: “Anything else?”**  (Circle responses under the “unprompted colomn)  **After PROBING, read out the list of symptoms that were not mentioned and circle responses under the “prompted column** | \|  \| **Unprompted** \| **Prompted** \| \| **Most Serious Episode (Q604)** \| \| --- \| --- \| --- \| --- \| --- \| \|  \| Yes \| Yes \| No \| Check Box \| \| Fever \| 1 \| 2 \| 3 \|  \| \| Unable to suckle/feed \| 1 \| 2 \| 3 \|  \| \| Difficult/fast breathing \| 1 \| 2 \| 3 \|  \| \| Diarrhea \| 1 \| 2 \| 3 \|  \| \| Convulsions \| 1 \| 2 \| 3 \|  \| \| Persistent vomiting \| 1 \| 2 \| 3 \|  \| \| Yellow palms/soles/eyes \| 1 \| 2 \| 3 \|  \| \| Lethargy \| 1 \| 2 \| 3 \|  \| \| Unconscious \| 1 \| 2 \| 3 \|  \| \| Red/discharging eyes \| 1 \| 2 \| 3 \|  \| \| Skin pustules \| 1 \| 2 \| 3 \|  \| \| Skin around cord red \| 1 \| 2 \| 3 \|  \| \| Pus from Cord \| 1 \| 2 \| 3 \|  \| \| Other \| 1 \|  \|  \|  \| \| Specify \| | |
| 603 | How many episodes of illness did (NAME/BABY) have? | Number [___\|___] |  |
| 604 | **If more than one episode of illness, Identifywhat mother felt was most serious episode. check the appropriate box in the last column inthe table for Q602** | |  |
| 605 | MOST SERIOUS ILLNESS  How old was (NAME/BABY) when the problem started?  If less than 1 day, record hours. If less than 1 week, record days. Otherwise record weeks. | \| Hours………………. \|  \|  \| \| --- \| --- \| --- \| \| Days……………….. \|  \|  \| \| Weeks……………… \|  \|  \| \| Don’t know…...........9 \|  \|  \| |  |

| 606 | How was (name) treated for this illness at home? | YES NO   1. By giving drugs………………1 2 2. By giving herbs………………1 2 3. By bringing health   provider to home…..1 2   1. By taking advice of   health provider……1 2   1. No treatment…………………1 2 2. Other…………………………….…1 2   Other specify ________________________________ |  |
| --- | --- | --- | --- |
| 607 | How much time after illness started was care initialized at home? | Hours [___\|___]  Days [___\|___]  No care given at home…………………….…………….96  don’t know................................................................... 98 |  |
| 608 | Did you seek advice or treatment for the illness outside the home? | Yes 1  No 2  Don’t Know 9 | 610   \|  \| \| --- \| \|  \| |

| 617 | Why didn’t you seek care for your neonate outside your home?  If the respondent says, respected ‘FAMILY MEMBERS DID NOT ALLOW’, probe to identify who that family member is: husband? Mother? Mother-in-law? Father? Father-in-law? Grand mother/grand father?  Specify | Mentioned: Yes No   1. Expecting self resolution of the illness…1 2 2. Health facility too far/no transportation..1 2 3. Cost of treatment service high………….1 2 4. Don’t trust facility/poor quality of   care……………1 2   1. Respected family members did   not allow ……….1 2   1. The traditional birth attendant   didn’t allow………1 2   1. Not customary to seek care outside home   after childbirth……1 2   1. Other …….…………………………… .1 2   Specify _______________________________   1. No reason given…………..…………….1 2 | 618  618  618  618  618  618  618  618  618 |
| --- | --- | --- | --- |

| 610 | How much time after illness started was (name) brought outside the home for care? | Hours [___\|___]  Days [___\|___]  don’t know .......................................................... 99 |  |
| --- | --- | --- | --- |

| 611 | From where did you seek care?  Anywhere else?  Circle all providers mentioned, but do NOT prompt with any suggestions. | YES NO  PUBLIC SECTOR   1. Govt. hospital 1 2 2. Govt. health center 1 2 3. Govt. health post 1 2   PRIVATE MEDICAL SECTOR   1. Private hospital/clinic 1 2 2. Private pharmacy 1 2 3. NGO health facility….…1 2   OTHER SOURCE   1. Relative or friend 1 2 2. Shop 1 2 3. Traditional practitioner 1 2 4. Other 1 2   (specify)_____________________________________ |  |
| --- | --- | --- | --- |
| 612 | **CHECK 610** | \| Two or more codes circled ……………1 \| \| --- \| \|  \|   Only one code circled…………………2 | 613  614 |
| 613 | Where did you first seek advice or treatment? | YES NO  PUBLIC SECTOR   1. Govt. hospital 1 2 2. Govt. health center 1 2 3. Govt. health post 1 2   PRIVATE MEDICAL SECTOR   1. Private hospital/clinic 1 2 2. Private pharmacy 1 2 3. NGO health facility….…1 2   OTHER SOURCE   1. Relative or friend 1 2 2. Shop 1 2 3. Traditional practitioner 1 2 4. Other 1 2   (specify)_____________________________________ |  |

| 614 | How did you take (name) to the hospital/clinic/care provider? | Taxi…………………………………….1  Bus…………………………………….2  Bicycle…………………………………3  Motor Cycle……………………………4  Horse/Donkey…………………………5  Horse/Donkey Cart…………………..6  On foot…………………………………7  Other …………………………………..8  Specify_____________________________ |  |
| --- | --- | --- | --- |
| 615 | Was it difficult to find the transport? | Yes………………………………………….…1  No……………………………………………..2  Don’t Know…………………………………...9 |  |
| 616 | How much time did it take to go there? | Minutes [_____]______]  Hours [_____]______]  Don’t Know…………………………….98 |  |
| 617 | On your way to the health facility (other), what did you do to care for your baby? | Mentioned: Yes No   1. Skin-to-skin…………….………………1 2 2. Kept baby bundled..……………..……..1 2 3. Nothing…………………………………1 2 4. Breastfed………………………………..1 2 5. Other……………………………………1 2   Specify______________________________  Don’t Know…………………………….8 | 618 |
| 618 | What medicine/local remedy was given to your child for the illness? | Mentioned: Yes No   1. Antibiotics..……….…………………1 2 2. Paracetamol……………………..……1 2 3. Gave herbals/other traditionally 4. prepared substances…….……………1 2 5. Other ……………………..………….1 2   Specify______________________________   1. Nothing………………………………1 2 |  |

| 619 | Now I would like to know how frequently your baby was breastfed during the illness. Was he/she breastfed less than usual, about the same or more than usual frequency? | Less than usual…………………... 1  Same as usual………………... …..2  More than usual…………… …..…3  Nothing to drink ………………….4  Don't know . . . . . …………….......9 |  |
| --- | --- | --- | --- |

| **Section 7: Nutrition** | | | |
| --- | --- | --- | --- |
| 701 | Are you still breastfeeding (NAME OF CHILD)? | Yes . . . . . . . . . . . . . . 1  No . . . . . . . . . . . . . . 2 | 704 |
| 702 | How many times did you breastfeed (name) last night between sunset and sunrise?  If answer is not numeric, probe for approximate number. | \| Number of nighttime feedings…… \|  \|  \| \| --- \| --- \| --- \|   Can’t remember …………………………………….98 |  |
| 703 | How many times did you breastfeed yesterday during the daylight hours?  If answer is not numeric, probe for approximate number. | \| Number of daytime feedings…… \|  \|  \| \| --- \| --- \| --- \|   Can’t remember …………………………………….98 |  |
| 704 | For how many months did you breastfeed (NAME)? | Months. . ………  Don’t know. .... 9 |  |
| 705 | Did (NAME) drink anything from a bottle with a nipple yesterday or last night? | \| Yes…………………… \| 1 \| \| --- \| --- \| \| No…………………… \| 2 \| \| Don’t know…………. \| 9 \| |  |
| **Now I would like to ask you about liquids or foods**  **(NAME) had yesterday during the day or at night, either separately or combined with other foods** | | | |
| 706 | Did (NAME) drink:  Plain water?  Commercially produced infant formula?  Any other milk as powdered or fresh animal milk?  Fruit juice?  Tea or coffee?  Any other liquids? | \|  \| Yes \| No \| DK \| \| --- \| --- \| --- \| --- \| \| 1. Plain water…… \| 1 \| 2 \| 8 \| \| 1. Formula………. \| 1 \| 2 \| 8 \| \| 1. Milk ………… \| 1 \| 2 \| 8 \| \| 1. Juice………….. \| 1 \| 2 \| 8 \| \| 1. Tea/coffee… \| 1 \| 2 \| 8 \| \| 1. Other liquids… \| 1 \| 2 \| 8 \| | |

|  |  |  |  |
| --- | --- | --- | --- |
| T2 | **Thank the mother for her time and end the interview**  **Time at End of Interview** | _____:_____ | **END INTERVIEW** |
